# Classification of Diabetic Foot Ulcers Using Class Knowledge Banks

**DOI:** 10.3389/fbioe.2021.811028

**Published:** 2022-02-28

**Authors:** Yi Xu, Kang Han, Yongming Zhou, Jian Wu, Xin Xie, Wei Xiang

**Affiliations:** ^1^ Shanghai TCM-Integrated Hospital, Shanghai University of Traditional Chinese Medicine, Shanghai, China; ^2^ College of Science and Engineering, James Cook University, Cairns, QLD, Australia; ^3^ Yueyang Hospital of Integrated Traditional Chinese Medicine and Western Medicine, Shanghai University of Traditional Chinese Medicine, Shanghai, China; ^4^ School of Engineering and Mathematical Sciences, La Trobe University, Melbourne, VIC, Australia

**Keywords:** diabetic foot ulcer, image recongnition system, deep learning, infection and ischemia classification, knowledge learning

## Abstract

Diabetic foot ulcers (DFUs) are one of the most common complications of diabetes. Identifying the presence of infection and ischemia in DFU is important for ulcer examination and treatment planning. Recently, the computerized classification of infection and ischaemia of DFU based on deep learning methods has shown promising performance. Most state-of-the-art DFU image classification methods employ deep neural networks, especially convolutional neural networks, to extract discriminative features, and predict class probabilities from the extracted features by fully connected neural networks. In the testing, the prediction depends on an individual input image and trained parameters, where knowledge in the training data is not explicitly utilized. To better utilize the knowledge in the training data, we propose class knowledge banks (CKBs) consisting of trainable units that can effectively extract and represent class knowledge. Each unit in a CKB is used to compute similarity with a representation extracted from an input image. The averaged similarity between units in the CKB and the representation can be regarded as the logit of the considered input. In this way, the prediction depends not only on input images and trained parameters in networks but the class knowledge extracted from the training data and stored in the CKBs. Experimental results show that the proposed method can effectively improve the performance of DFU infection and ischaemia classifications.

## 1 Introduction

The diabetic foot ulcer (DFU) is a complication of diabetes with high incidence Armstrong et al. (2017). According to the estimation of the International Diabetes Federation Atlas et al. (2015), 9.1 million to 26.1 million people with diabetes develop foot ulcers each year in the world. For people with diabetes, the presence of DFU can result in amputation and even increase the risk of death Walsh et al. (2016). Identifying whether the DFU is infection and ischaemia is important for its assessment, treatment, and management Jeffcoate and Harding (2003), where the infection is defined as bacterial soft tissue or bone infection in the DFU and ischaemia means inadequate blood supply Goyal et al. (2020). Classification of DFU infection and ischaemia by computerized methods is thus a critical research problem for automatic DFU assessment.

Traditional methods for diagnosis of DFU employ hand-crafted features followed by a classifier Veredas et al. (2009); Wannous et al. (2010); Wang et al. (2016). However, research in literature has shown that learned features by deep neural networks are more effective than traditional hand-crafted features LeCun et al. (2015). Extensive research has been done to increase the performance of computerized automatic medical image classification Litjens et al. (2017), where methods based on deep learning LeCun et al. (2015) are very popular in this field because they perform significantly better than other techniques Li et al. (2014); Kumar et al. (2016); Goyal et al. (2020); Wang et al. (2020); Cao et al. (2021).

The most widely used deep learning method in medical image classification is the convolutional neural network (CNN) Gulshan et al. (2016); Albawi et al. (2017). CNNs can effectively extract useful features for image classification He et al. (2016); Tan and Le (2019), object detection Redmon et al. (2016); Zhao et al. (2019), image segmentation Chen et al. (2018); Hesamian et al. (2019); Xiao et al. (2021) and many other vision tasks LeCun et al. (2015). With the availability of large-scale training data and high-performance modern GPUs and ASICs, methods based on CNNs have greatly improved the accuracy of image classification. Popular CNNs for general image classification tasks include AlexNet Krizhevsky et al. (2012), VGG Simonyan and Zisserman (2014), ResNet He et al. (2016), and EfficientNet Tan and Le (2019). These networks usually serve as the backbone of a medical image classification network, or directly apply to medical image classification by transfer learning with pre-trained parameters on large-scale datasets, e.g., ImageNet Deng et al. (2009). In practice, collecting and labeling medical images are costly. Transfer learning is thus an effective way to solve the problem of the lack of medical training data Shie et al. (2015); Shin et al. (2016); Cheplygina et al. (2019); Chen et al. (2020a).

Other emerging techniques for image classification include vision transformer Dosovitskiy et al. (2020); Touvron et al. (2020); Liu et al. (2021) and contrastive learning Wang et al. (2020); Jaiswal et al. (2021). Vision transformer methods are based on the attention mechanism, where an input image is split into small patches and the vision transformer can learn to focus on the most important regions for classification. Contrastive learning usually performs in an unsupervised way, where the network learns to minimize intra-class distance and maximize inter-class distance. The networks trained by contrastive learning perform well on the subsequent tasks like image segmentation but their classification accuracies are still inferior to those of state-of-the-art supervised methods.

However, existing medical image classification networks do not explicitly consider class knowledge in the training data when performing prediction. The training of existing networks involves the optimization of network parameters, where the class knowledge in the training data is extracted implicitly. In the testing, the trained networks process an input image into a high dimensional representation through trained parameters, where the class knowledge in the training data is not explicitly involved in the pipeline. To better utilize the class knowledge in the training data, we propose class knowledge banks (CKBs) that can effectively extract class knowledge from the training data, and the extracted class knowledge can directly participate in the prediction process. A CKB consists of many trainable units that can represent class knowledge from different perspectives. The average similarity between a representation extracted from an input image and knowledge units in the CKB can be used as a class probability. In this way, the class knowledge in the training data is explicitly utilized. Besides, the proposed CKB method can handle class imbalance as each class is given the same importance in CKBs. As a result, the network with the CKB is able to achieve state-of-the-art classification performance in the DFU image dataset (Goyal et al. (2020)). In summary, we make the following contributions:

• We propose a class knowledge bank (CKB) method that can explicitly and efficiently extract and utilize class knowledge in the training data.
• We show that the proposed CKB is good at handling class imbalance in the DFU image classification dataset.

The remainder of the paper is organized as follows. We first briefly review the related work in Section 2. Then we describe the proposed method in detail in Section 3. Sections 4, 5 present experimental results and discussions. We conclude the paper in Section 6.

## 2 Related Work

In this section, we briefly review the related work on image classification, including convolutional neural networks, vision transformers, and contrastive learning.

### 2.1 Convolutional Neural Networks

Convolutional neural networks (CNNs) are the most widely used technique for image classification LeCun et al. (2015). CNNs utilize multiple convolutional kernels in each layer and multiple layers to extract and process features from low levels to high levels. Since the success of AlexNet Krizhevsky et al. (2012) in image classification in 2012, a lot of methods based on CNN have been proposed to tackle this problem, and the performance of image classification on large datasets, e.g., ImageNet Deng et al. (2009), has been significantly improved. Many medical image classification methods are based on CNNs Li et al. (2014); Kumar et al. (2016); Anwar et al. (2018); Yadav and Jadhav (2019); Feng et al. (2020). Typical networks for image classification include VGG Simonyan and Zisserman (2014), ResNet He et al. (2016), Densenet Huang et al. (2017), Efficientnet Tan and Le (2019), and RegNet Radosavovic et al. (2020). These networks follow the structure of deep CNNs (to extract feature) and fully-connected (FC) layers (to predict classes). After training, the prediction depends on the input image and parameters in CNNs and FC layers, without explicit use of the class knowledge in the training data.

### 2.2 Vision Transformers

The transformer model was firstly proposed for natural language processing Vaswani et al. (2017). The model uses an attention mechanism Gao et al. (2021); Wang et al. (2017) to capture the correlation within tokens and learns to focus on important tokens. Dosovitskiy et al. (2020) first applied the transformer to image classification and achieved an even better performance than CNNs on the ImageNet dataset. Such a model is called vision transformer, where an input image is divided into patches and these patches are regarded as tokens to feed into the network. The vision transformer can learn to focus on important regions by the attention mechanism to predict class labels. Vision transformers have also been applied in medical image classification Dai et al. (2021). Furthermore, knowledge distillation Hinton et al. (2015); Wei et al. (2020) from the model based on CNN is shown to be effective in improving the performance of the vision transformer Touvron et al. (2020). Instead of simply regarding image patches as tokens, Yuan *et al.* proposed a tokens-to-token (T2T) method to better tokenize patches with the consideration of image structure Yuan et al. (2021). The T2T method achieves better accuracy using fewer parameters compared with the vanilla vision transformer Dosovitskiy et al. (2020).

### 2.3 Contrastive Learning

Contrastive learning aims at learning effective representations by maximizing the similarity between positive pairs and minimizing the similarity between negative pairs Jaiswal et al. (2021). It usually performs in a self-supervised manner, where positive pairs are from different augmentations of the same sample and negative pairs are simply different samples. SimCLR constructs contrastive loss by a large batch size, e.g., 4,096, to fully explore the similarity in negative pairs Chen et al. (2020b). Followed by SimCLR, SimCLRv2 achieves a better performance than SimCLR by leveraging bigger models and deeper projection head Chen et al. (2020c). Since contrastive learning requires a large number of representations of negative pairs, He *et al.* utilized a queue to store the representations of samples and updated the queue via a momentum mechanism He et al. (2020), which is shown to be more effective than sampling representations from the last epoch Wu et al. (2018). Although these contrastive learning methods achieve good performance on image classification by fine-tuning with few labeled samples, their performances are still inferior to those of state-of-the-art supervised methods.

Existing image classification networks do not explicitly take the class knowledge in the training data into account when performing prediction. To explicitly leverage class knowledge in the training data, we propose the so-called class knowledge bank method that is able to extract class knowledge from the training data, and the extracted class knowledge can directly participate in the prediction process.

## 3 Methods

### 3.1 Proposed Network Structure

**FIGURE 1:**
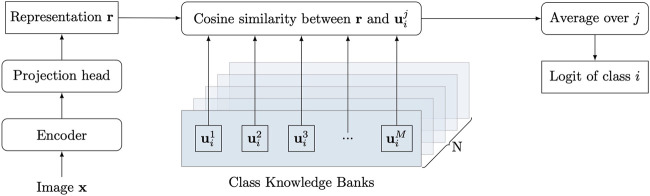
Overview of the proposed network. The encoder and projection head embed the input image x into a representation r . The average similarity between the representation r and the unit $u_{i}^{j}$ in the class knowledge bank (CKB) **C**_*i*_ is measured as the logit of **x** for class i. The CKBs are parameterized by a matrix of dimension *N* × *M* × *D*, where *N* is the number of classes, *M* is the number of units in each CKB, and D denotes the dimension of a unit. The CKBs are randomly initialized and can be trained through back-propagation.

Given an input medical image x , the goal of image classification is to produce its class y∈{0,1,…,N−1} , where *N* is the number of classes. Existing deep neural networks for image classification usually extract discriminative features (representations) through a layer-by-layer structure, and directly yield the class from extracted features by multilayer perceptrons (MLPs). We introduce the class knowledge bank into the traditional pipeline to enable explicit utilization of class knowledge in the training data. As shown in Figure 1, the input image x is first fed to an encoder and then a projection head to extract a high-level representation $r\in R^{D}$

$$\begin{array}{cc} r_{0} & =Encoder\left(x\right)\\ r & =Projection\left(r_{0}\right) \end{array}$$ (1)

where *D* is the dimension of the extracted representation. The projection head is introduced for two main purposes. Firstly, it can transfer the representation extracted by the encoder to a space that is suitable for contrastive learning, and thus improves the quality of the representation. Secondly, the representation from the encoder does not contain information specific to diabetic foot images as the encoder is pre-trained on a large-scale natural image dataset and froze when training the proposed network. The projection head can learn specific useful information from the diabetic foot dataset to improve classification performance. The extracted representation r is then used to compute the similarity with units in CKBs. The computed similarity can be regarded as the logits and used to build a contrastive loss to train the network.

### 3.2 Class Knowledge Bank

Each class of images has its properties, such as color and structure, which can be used to distinguish them from other classes of images. The knowledge of a class should contain these properties from different perspectives to comprehensively describe the class. The CKB method is proposed to achieve this goal and one CKB is designed to represent the knowledge of one class. A CKB consists of a number of units that can represent class knowledge from different perspectives. Each unit in the CKB is of the same size as the extracted representation r and there are *M* units in a CKB. The size of a CKB is thus *M × D*. For image classification with *N* classes, we need *N* CKBs to store all the class knowledge. A CKB for class *i* can be represented as **C**_*i*_

$$C_{i}=\left\{u_{i}^{1},u_{i}^{2},u_{i}^{3},\ldots ,u_{i}^{M}\right\}$$ (2)

where u denotes the unit in the CKB. The average similarity *s*_*i*_ between the **C**_*i*_ and the extracted representation r can be measured by the mean similarity across units

$$s_{i}=\frac{1}{M}\underset{j}{\sum }\cos\left(u_{i}^{j},r\right)$$ (3)

where cos (⋅, ⋅) is the cosine similarity

$$\cos\left(u_{i}^{j},r\right)=\frac{u_{i}^{j}\cdot r}{∥u_{i}^{j}∥∥r∥}.$$ (4)

A large *s*_*i*_ indicates the representation is close to the class *i*, which means the input x has a high probability of class *i*.

The measured similarities between the representation and the CKBs can be seen as the logits of the input image, and thus can be used to compute probabilities of classes through softmax function

$$p_{i}=\frac{\exp\left(s_{i}\right)}{\sum_{k=0}^{N-1}\exp\left(s_{k}\right)}$$ (5)

where *p*_*i*_ is the probability of class *i*. Based on the similarities, we define the following contrastive loss

$$\begin{array}{cc} L_{CON}\left(C,r,y\right)= & -\frac{1}{M}\underset{j}{\sum }\cos\left(u_{y}^{j},r\right)\\ & +\frac{1}{N-1}\underset{i\neq y}{\sum }\frac{1}{M}\underset{j}{\sum }\cos\left(u_{i}^{j},r\right). \end{array}$$ (6)

Label y serves as the index of the correct class. Minimizing this contrastive loss is equivalent to maximizing the similarity between r and units in the correct CKB, and to minimizing the similarity between **r** and units in other CKBs. The final training loss is a combination of the above contrastive loss and cross-entropy loss $L_{CEL}$ :

$$L=L_{CON}\left(C,r,y\right)+L_{CEL}\left(s,y\right)$$ (7)

where **s** = {*s*_0_, *s*_1_, … , *s*_*N*−1_} are logits (represented by averaged similarities in (3)) of the input.

The units in CKBs are randomly initialized and then optimized through back-propagation. The network will try to extract the class knowledge into the CKBs with the objective of minimizing the designed contrastive loss in (6). In this way, the proposed CKB method is more effective in utilizing knowledge in the training data than existing contrastive learning methods, e.g., end-to-end mechanism Oord et al. (2018), memory bank Wu et al. (2018) and momentum contrast He et al. (2020). This effectiveness is mainly derived from two aspects. Firstly, the proposed CKBs do not rely on a large number of specific samples. Instead, CKBs can learn to extract class knowledge and represent them by the units in the CKBs. Since the CKBs are optimized on the whole training dataset, they contain more comprehensive knowledge than some specific samples. Secondly, a small number of, e.g., 64, units in a CKB can represent the knowledge of one class very well, which can greatly reduce the computational complexity and memory usage compared with existing contrastive learning methods that usually require thousands of samples in one training iteration.

**FIGURE 2:**
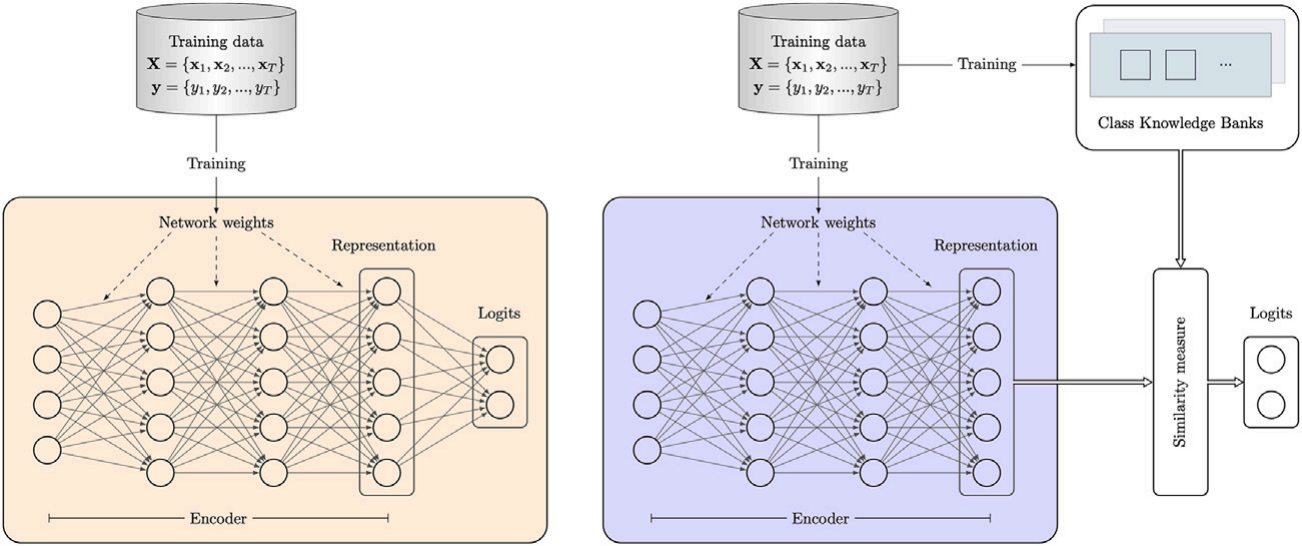
Comparison between common classification networks and the network with the proposed class knowledge banks. A common image classification network on the left is trained on the training data. After training, class knowledge in the training data only functions through the network weights. By contrast, except for the network weights, the proposed class knowledge banks can learn class knowledge from the training data and store the knowledge. The learned knowledge in the CKBs participates in the classification process by measuring the similarity between the CKBs and the representation of the input.

Figure 2 compares the proposed method and existing popular image classification methods. In existing image classification networks, parameters are mainly weights that are trained via back-propagation using the training data. The classification is achieved by directly predicting class logits from the discriminative representation extracted from the encoder. In such process, class knowledge in the training data is not explicitly utilized as the network is trained to focus on extracting more discriminative representation from the input. As shown in Figure 2, the network with the proposed CKBs has a different pipeline of producing class logits. The CKBs learn and represent class knowledge through units parameterized by vectors. Then the learned class knowledge in the CKBs explicitly participates in the classification process by measuring the similarity between the units in the CKBs and the representation of the input. In this way, the class knowledge in the training data not only implicitly functions through network weights but explicitly works in the form of class similarity.

### 3.3 Encoder and Projection Head Structures

**FIGURE 3:**
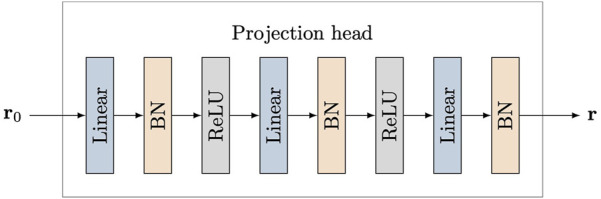
Structure of projection head. Three linear layers are used and the first two linear layers are followed with batch normalization (BN) and ReLU activation. The input of the projection head is the representation **r**_0_ extracted from the encoder, and its output is the transformed representation **r** used for contrastive learning.

The encoder is concerned with extracting a discriminative representation from an input image. Training the network with an encoder from scratch on medical image datasets is not effective since medical datasets are usually comparatively small. Thus, we employ a pre-trained image classification network Touvron et al. (2020) that is trained on ImageNet as the encoder in our proposed network. This strategy is shown to be very effective for many medical image processing tasks when training datasets are small Shin et al. (2016); Goyal et al. (2020); Chen et al. (2021). We further introduce a multilayer perceptron (MLP) projection head as in Chen et al. (2020c) to transform the output representation from the encoder into a suitable space for contrastive learning. As shown in Figure 3, the input of the projection head is the representation **r**_0_ extracted from the encoder, and its output is the transformed representation **r** used for the following contrastive learning. The MLP projection head includes three linear layers and the first two linear layers are followed by batch normalization (BN) Ioffe and Szegedy (2015) and ReLU activation Nair and Hinton (2010). The output of the last linear layer is only processed by batch normalization without ReLU activation.

## 4 Results

**FIGURE 4:**
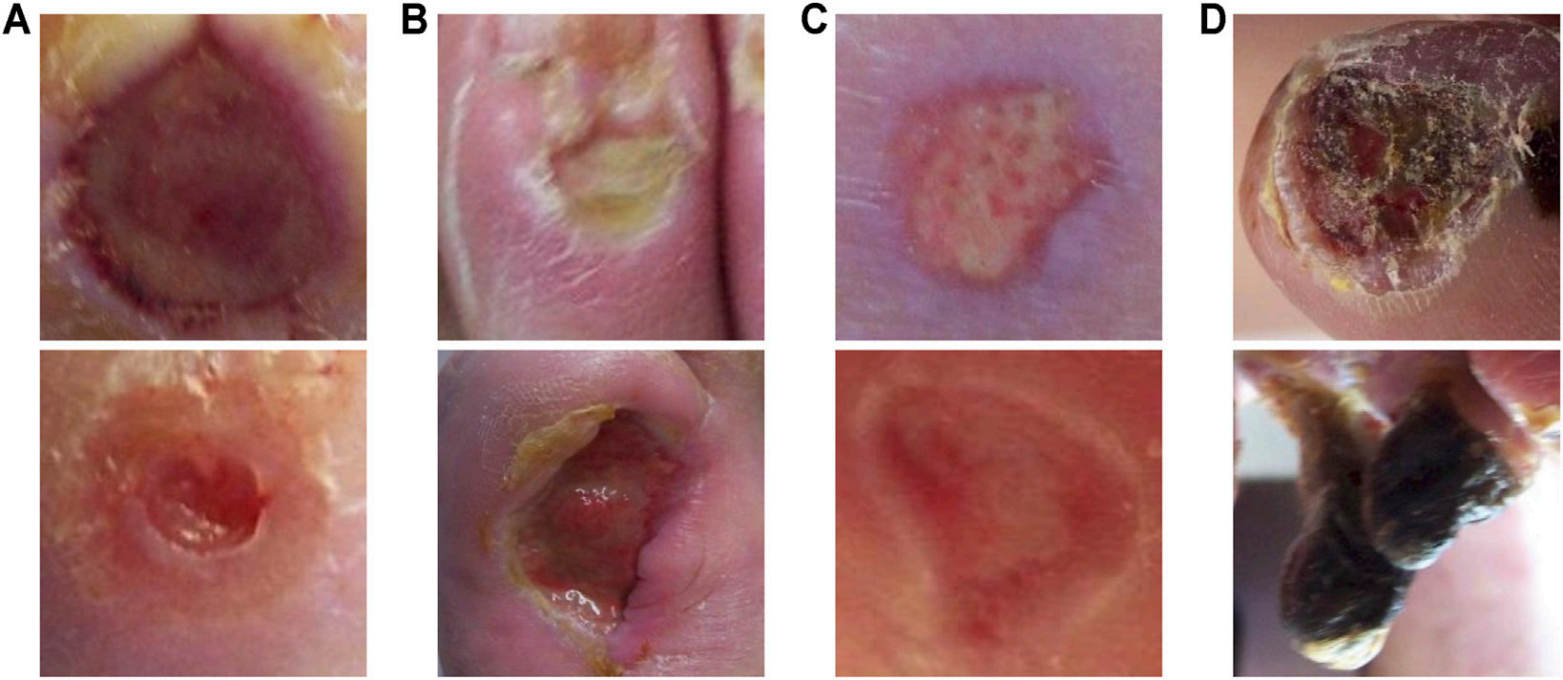
Sample images from the DFU dataset Goyal et al. (2020). **(A)** are non-infection images, **(B)** are infection images, **(C)** are non-ischaemia images and **(D)** are ischaemia images.

We use the diabetic foot ulcer (DFU) dataset in Goyal et al. (2020) to evaluate the performance of the proposed method. The DFU dataset includes ischaemia and infection parts that were collected from the Lancashire Teaching Hospitals. There are 628 non-infection and 831 infection cases, and 1,249 non-ischaemia and 210 ischaemia cases in the dataset. It can be observed that class imbalance exists in this dataset. The collected images were labeled by two healthcare professionals and augmented by the natural data augmentation method which extracts region of interest (ROI) ulcers by a learn-based ROI localization method Goyal et al. (2018). After augmentation, the ischaemia and infection parts include 9,870 and 5,892 augmented image patches, respectively. Figure 4 shows samples of infection and ischaemia images from this dataset. We use 5-fold cross-validation and report on average performance and standard deviation.

The proposed method is implemented by the deep learning library Pytorch Paszke et al. (2017). We utilize the AdamW Loshchilov and Hutter (2017) algorithm as the optimizer to train models. The AdamW improves the generalization performance of the commonly used Adam algorithm Kingma and Ba (2014). The learning rate and weight decay are initialized to be 5e-4 and 0.01, respectively. The step learning rate scheduler is employed with the step size of 2 and the decay factor of 0.6. We use the batch size of 64 and train models in 20 epochs. Several popular image classification models are used for performance comparisons, including CNN-based ResNet He et al. (2016), RegNetY Radosavovic et al. (2020), EfficientNet Tan and Le (2019), contrastive learning-based MoCo He et al. (2020), and vision transformer-based DeiT Touvron et al. (2020). The input images are resized to the resolution of 224 × 224 for all methods for fair comparison.

To investigate whether larger models can lead to better performance, we evaluate the performance of the above models with different layers. Small and base DeiT models are denoted as DeiT-S and DeiT-B. For fair comparisons, all the competing methods use three linear layers with dimension 512 (first and second layers are followed by ReLU) as their classifiers, where the objective of the classifiers is to yield the logits. The objective of the projection head in our method is to produce discriminative representations. The number of units in each CKB is 64. Batch normalization (BN) is not applied in the MLP classifiers for comparison methods, since BN degrades these networks’ performances. For all methods, we use the models pre-trained on ImageNet and freeze their parameters except for the parameters in the MLP classifiers, MLP projection head, and CKBs. We find freezing the pre-trained parameters leads to better performance than fine-tuning the whole network. For DeiT with knowledge distillation, there are two classifiers or projection heads that process the class token and distillation token, and the final prediction is the sum of two logits. We use accuracy, sensitivity, precision, specificity, F-measure, and area under the ROC curve (AUC) to measure the performance of the classification models.

Table 1 presents the DFU infection classification performances of various methods. As shown in Table 1, larger CNN models usually produce better results. The F-measure and AUC score of ResNet-152 are superior to those of ResNet-101. Similar results are also observed for RegNetY, where RegNetY-16GF achieves better performances than RegNetY-4GF and RegNetY-8GF. However, the performance differences for EfficientNet with different sizes are not significant, and the large model even performs slightly worse than small models. MoCo with the backbone of ResNet-50 performs better than the vanilla ResNet-50 for infection classification, showing that the contrastive learning method helps the network learns more discriminative representations for image classifications. Vision transformer-based DeiT models trained with knowledge distillation (denoted as DeiT-S-D and DeiT-B-D) perform better than CNN models. This is reasonable as DeiT-B-D is shown to perform better than the comparison CNN models on ImageNet classification task Touvron et al. (2020). The superior performance of DeiT-B-D when transferred for the task of diabetic foot infection classification demonstrates its robustness. We also observe a phenomenon similar with Touvron et al. (2020) that knowledge distillation can significantly improve the performance of DeiT. For example, the F-measure and AUC score of DeiT-B-D are 76.72 and 83.26, which are better than those of DeiT-B (F-measure 74.91 and AUC 81.58) by large margins. The performance improvements of knowledge distillation for DeiT may be due to the inherited inductive bias from the CNN-based teacher model, e.g., RegNet Radosavovic et al. (2020), where DeiT mainly consists of multilayer perceptrons and attention modules.

**TABLE 1:** Performance of binary classification on the DFU infection dataset.

Network	Accuracy	Sensitivity	Precision	Specificity	F-measure	AUC score
ResNet-18	74.20 ± 1.25	76.66 ± 2.82	73.08 ± 4.13	71.98 ± 2.98	74.72 ± 2.08	82.23 ± 1.26
ResNet-50	73.79 ± 1.31	76.96 ± 3.13	72.33 ± 2.93	70.75 ± 0.92	74.50 ± 1.98	81.44 ± 1.60
ResNet-101	74.63 ± 0.98	75.97 ± 0.88	73.88 ± 2.65	73.26 ± 1.37	74.89 ± 1.63	82.52 ± 0.81
ResNet-152	74.82 ± 1.02	76.80 ± 0.67	73.82 ± 2.42	72.82 ± 1.95	75.25 ± 1.17	82.78 ± 0.85
RegNetY-4GF	73.63 ± 1.50	75.92 ± 1.97	72.57 ± 2.72	71.37 ± 2.30	74.16 ± 1.53	81.33 ± 1.54
RegNetY-8GF	74.85 ± 1.51	76.72 ± 2.18	73.91 ± 3.18	73.00 ± 2.66	75.24 ± 1.89	81.90 ± 1.60
RegNetY-16GF	75.41 ± 0.95	77.42 ± 1.48	74.44 ± 2.61	73.51 ± 1.82	75.85 ± 0.75	83.02 ± 1.28
EfficientNet-B0	73.95 ± 1.06	77.81 ± 2.48	72.23 ± 3.51	70.19 ± 3.00	74.83 ± 1.75	81.77 ± 1.01
EfficientNet-B2	73.85 ± 1.21	77.64 ± 1.15	72.13 ± 3.34	70.09 ± 2.88	74.73 ± 1.70	81.58 ± 0.90
EfficientNet-B4	73.61 ± 1.17	77.24 ± 2.28	72.05 ± 3.78	70.16 ± 3.07	74.46 ± 1.66	81.70 ± 1.08
EfficientNet-B6	73.43 ± 0.54	75.77 ± 1.85	72.40 ± 3.30	71.29 ± 1.69	73.97 ± 1.14	80.98 ± 1.10
EfficientNet-B7	72.79 ± 1.53	72.14 ± 3.10	73.10 ± 2.60	73.43 ± 3.16	72.54 ± 1.67	80.08 ± 1.26
MoCo	74.97 ± 2.01	74.06 ± 1.75	75.47 ± 4.37	75.96 ± 4.08	74.68 ± 2.22	82.77 ± 1.46
DeiT-S	73.65 ± 0.64	77.22 ± 1.79	72.02 ± 3.18	70.14 ± 1.89	74.47 ± 1.62	80.90 ± 0.95
DeiT-B	73.97 ± 0.83	78.09 ± 2.23	72.12 ± 3.40	69.97 ± 2.42	74.91 ± 1.63	81.58 ± 1.35
DeiT-S-D	73.98 ± 1.97	78.06 ± 2.07	72.21 ± 4.28	70.09 ± 3.81	74.93 ± 2.23	81.51 ± 1.81
DeiT-B-D	75.82 ± 1.96	**79.96 ± 2.88**	73.86 ± 3.33	71.88 ± 2.14	76.72 ± 2.11	83.26 ± 2.36
CKB-DeiT-S-D	75.18 ± 1.27	76.91 ± 2.15	74.36 ± 3.79	73.54 ± 3.44	75.53 ± 1.78	82.66 ± 1.28
CKB-DeiT-B-D	**78.00 ± 0.93**	79.16 ± 1.74	**77.38 ± 2.68**	**77.00 ± 1.51**	**78.20 ± 0.94**	**84.78 ± 1.30**

**FIGURE 5:**
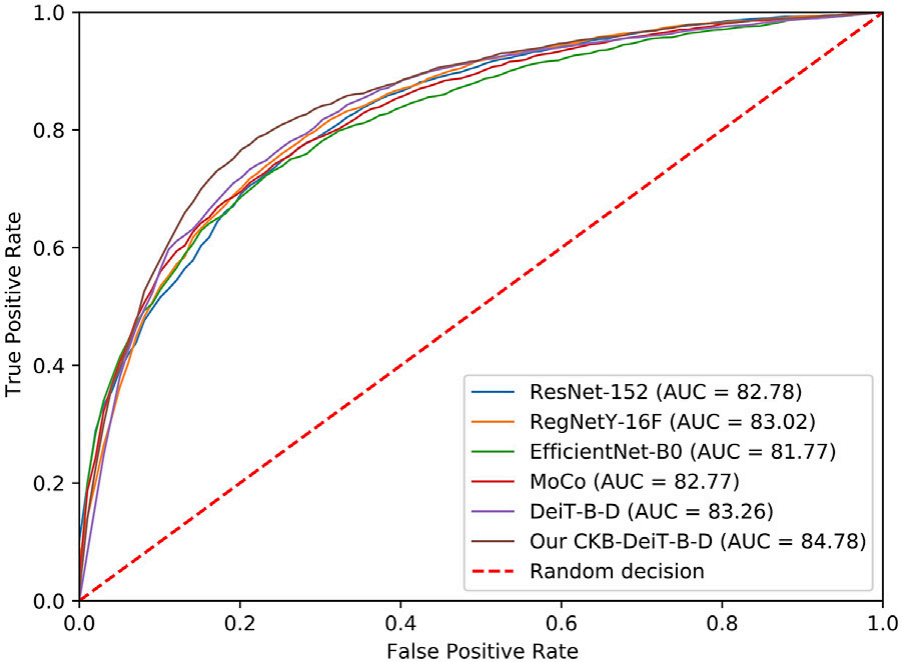
ROC curves of different methods for the DFU infection classification.

Furthermore, when distilled DeiT is used in conjunction with the proposed CKB, denoted by CKB-DeiT-B-D, further performance improvements are obtained, leading to the best performance for infection classification on all performance metrics except sensitivity. As can be seen from Table 1, the proposed CKB-DeiT-B-D performs better than the latest vision transformer DeiT-B-D, and significantly better than other comparison CNN-based methods in terms of all the reported metrics except sensitivity. For instance, the proposed CKB-DeiT-B-D achieves the best F-measure of 78.20 and the best AUC score of 84.78, which are better than the results of 76.72 and 83.26 achieved by the second-best DeiT-B-D, and significantly better than the results of 75.85 and 83.02 achieved by the CNN-based RegNetY-16GF. The proposed CKB significantly improves the precision and specificity of DeiT-B-D, e.g., improving precision from 73.86 to 77.38 and specificity from 71.88 to 77.00. Also, CKB-DeiT-S-D that combines the CKB with the small DeiT with knowledge distillation performs better than the vanilla DeiT-S-D. Although the proposed CKB-DeiT-B-D performs slightly worse in terms of sensitivity, the performance improvements on all the other metrics demonstrate the superiority of the proposed method. In Figure 5, we compare the ROC curves of the comparison methods. The methods that achieve the best AUC score over the networks with the same architecture but different layers are selected for comparison. It can be observed from Figure 5 that our proposed CKB-DeiT-B-D produces a better ROC curve than the comparison methods.

**FIGURE 6:**
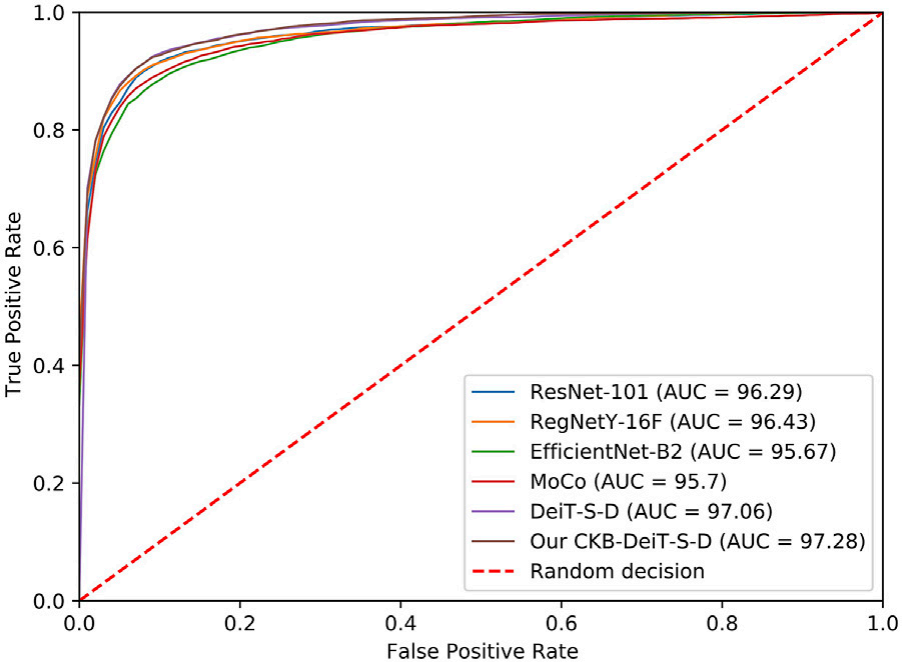
ROC curves of different methods for the DFU ischaemia classification.

The proposed method also achieves the best accuracy, sensitivity, F-measure, and AUC score on the DFU ischaemia dataset. As shown in Table 2, the performances of different methods on the DFU ischaemia dataset are better than their performances on the DFU infection dataset since the characteristics of ischaemia are more discriminative as shown in Figure 4. The precision and specificity of the proposed method are better than the CNN-based methods (ResNet, RegNetY, and EfficientNet) and contrastive learning method (MoCo) but inferior to the DeiT-B-D. The comparison methods all seem to produce high precision and specificity but significantly lower accuracy, sensitivity, and F-measure. The proposed CKB-DeiT-B-D produces more balanced results across all the reported metrics. The proposed CKB-DeiT-S-D achieves the best AUC score but the improvement of the ROC curve of our method shown in Figure 6 is not significant compared with DeiT-S-D. Overall, the proposed CKB using DeiT Touvron et al. (2020) as the encoder achieves the best infection and ischaemia classification performances in terms of most metrics.

**TABLE 2:** Performance of binary classification on the DFU ischaemia dataset.

Network	Accuracy	Sensitivity	Precision	Specificity	F-measure	AUC score
ResNet-18	88.30 ± 1.36	82.40 ± 3.48	93.23 ± 1.73	94.16 ± 1.03	87.43 ± 2.03	95.48 ± 0.89
ResNet-50	88.13 ± 1.77	81.46 ± 3.49	93.80 ± 1.42	94.76 ± 0.59	87.16 ± 2.26	95.09 ± 1.24
ResNet-101	89.95 ± 1.29	85.17 ± 2.50	94.10 ± 1.25	94.78 ± 0.55	89.38 ± 1.37	96.29 ± 1.17
ResNet-152	88.62 ± 2.18	82.45 ± 3.16	93.92 ± 2.22	94.84 ± 1.55	87.79 ± 2.42	95.58 ± 1.15
RegNetY-4GF	89.55 ± 0.89	83.64 ± 1.53	94.66 ± 1.53	95.41 ± 1.05	88.80 ± 1.34	95.92 ± 1.33
RegNetY-8GF	89.36 ± 1.23	83.64 ± 1.61	94.45 ± 0.60	95.13 ± 0.45	88.70 ± 0.80	95.59 ± 1.07
RegNetY-16GF	90.48 ± 1.01	85.54 ± 2.00	94.79 ± 1.54	95.40 ± 1.19	89.91 ± 1.25	96.43 ± 1.03
EfficientNet-B0	87.26 ± 1.74	79.07 ± 3.89	94.38 ± 1.11	95.38 ± 0.68	85.99 ± 2.29	94.81 ± 0.90
EfficientNet-B2	88.23 ± 0.71	81.17 ± 2.42	94.37 ± 1.46	95.20 ± 1.23	87.24 ± 1.21	95.67 ± 0.92
EfficientNet-B4	87.24 ± 1.77	79.11 ± 3.75	94.27 ± 1.30	95.31 ± 0.67	85.98 ± 2.33	94.46 ± 1.22
EfficientNet-B6	87.40 ± 2.38	80.57 ± 4.96	93.41 ± 0.95	94.34 ± 0.95	86.41 ± 2.50	94.53 ± 1.21
EfficientNet-B7	86.41 ± 2.40	78.57 ± 4.95	93.06 ± 0.90	94.21 ± 0.58	85.11 ± 2.94	94.64 ± 1.62
MoCo	89.74 ± 1.29	86.01 ± 3.24	92.92 ± 1.86	93.56 ± 1.53	89.28 ± 1.41	95.70 ± 1.01
DeiT-S	88.89 ± 2.13	82.35 ± 4.19	94.58 ± 1.08	95.38 ± 0.64	87.99 ± 2.54	96.45 ± 0.95
DeiT-B	89.10 ± 2.32	81.76 ± 5.02	95.84 ± 1.13	96.50 ± 0.80	88.13 ± 2.68	96.19 ± 1.49
DeiT-S-D	89.96 ± 1.88	83.44 ± 3.65	95.96 ± 1.06	96.58 ± 0.63	89.21 ± 1.96	97.06 ± 1.07
DeiT-B-D	89.69 ± 1.93	82.51 ± 3.48	**96.29 ± 0.63**	**96.88 ± 0.17**	88.83 ± 2.12	96.61 ± 1.01
CKB-DeiT-S-D	90.27 ± 1.90	84.09 ± 4.00	95.97 ± 1.41	96.59 ± 0.86	89.57 ± 2.04	**97.28** ± **0.91**
CKB-DeiT-B-D	**90.90** ± **1.74**	**86.09** ± **2.98**	95.00 ± 1.29	95.59 ± 0.71	**90.30** ± **1.83**	96.80 ± 1.16

## 5 Discussions

The main finding of this research is that better utilization of class knowledge in the training data can improve the performance of DFU image classifications. We have proposed an approach called class knowledge bank which can explicitly and effectively extract class knowledge from the training data and participate in prediction process in the testing. Experimental results have demonstrated the effectiveness of the proposed method in improving classification performances on both DFU infection and ischaemia datasets.

**FIGURE 7:**
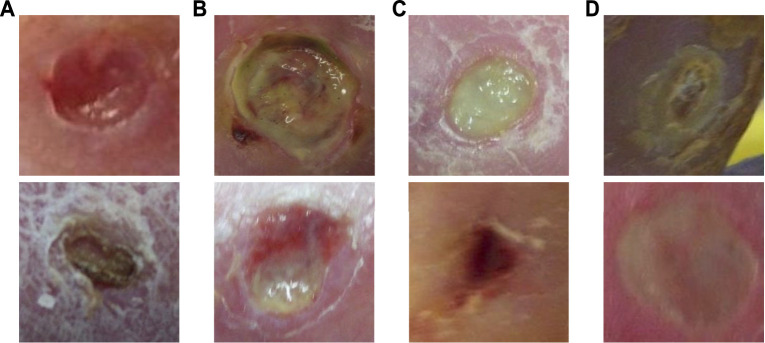
Examples of classification results of the proposed method on the infection dataset. **(A)** true negative cases, **(B)** true positive cases, **(C)** false negative cases and **(D)** false positive cases.

**FIGURE 8:**
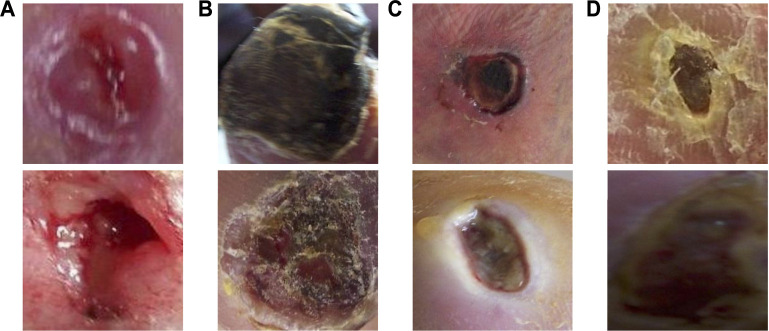
Examples of classification results of the proposed method on the ischaemia dataset. **(A)** true negative cases, **(B)** true positive cases, **(C)** false negative cases and **(D)** false positive cases.

Examples of classification results by the proposed method on the infection and ischaemia datasets are presented in Figures 7, 8, respectively. Correctly classified ulcer images (true negative and true positive) are shown to have discriminative visual characteristics, which are useful for image-based classifications. For instance, true negative non-infection cases in Figure 7A are clean and dry, while true positive infection cases in Figure 7B are full of yellow secretion. For ischaemia classification examples, it is observed from Figures 8A,B that color characteristic is very different between true negative and true positive cases. A close inspection of incorrectly classified cases in Figures 7C,D; Figures 8C,D suggests that many factors including lighting condition, size of ulcers, secretion, subtle characteristic and model’s ability can all affect classification results. This observation means that one needs to carefully consider these factors in real applications.

The proposed method is good at handling class imbalance than the comparison methods. As can be observed from Tables 1, 2, the specificity of the comparison methods is significantly worse than the sensitivity caused by the class imbalance on the infection dataset, while the proposed method can achieve high sensitivity and specificity simultaneously. Also, the proposed method produces more balanced sensitivity and specificity than the comparison methods on the ischaemia dataset. The advantage of the proposed method in handling imbalance data is derived from the structure of the class knowledge banks, where different CKBs have the same units which give the same importance to different classes.

The proposed classification network is based on a pre-trained powerful encoder as training a network from scratch on a relatively small medical image dataset is not efficient. This is a limitation of the proposed network because its performance relies on the pre-trained encoder. We believe that one can achieve better DFU classification performances without relying on a pre-trained encoder when more training data are available. Another limitation is that the proposed method does not consider the contrastive idea in samples in the training data and units in class knowledge banks. Incorporating this idea into the proposed method may further improve DFU classification performances. This paper verifies the performance of the proposed method on the DFU infection and ischaemia datasets. It will be interesting to extend this research to wider areas such as other medical image classification tasks, including binary or multi-class classification problems. The proposed method also has the potential to work as an incremental learning method as we can train additional class knowledge banks for incremental classes. Its performance and characteristics for incremental learning remain further investigation in the future.

## 6 Conclusion

In this paper, we proposed the method called the class knowledge banks (CKBs) which can effectively extract class knowledge from the training data and explicitly leverage the class knowledge in the testing. The proposed method is an alternative means to produce the logits instead of the usual linear classifiers in the literature. The CKBs leverage their units to extract and represent class knowledge from different perspectives and the similarities between the representation of the input and the corresponding CKBs can be regarded as the logits of the input. The CKB can be trained through back-propagation and be easily embedded into existing image classification models. Experimental results on the DFU infection and ischaemia datasets demonstrate the effectiveness of the proposed CKB in DFU image classifications.

## Data Availability

The original contributions presented in the study are included in the article/Supplementary Material, further inquiries can be directed to the corresponding author.
